# Supplementary material for: Carbonate Apatite and Hydroxyapatite Formulated with Minimal Ingredients to Deliver SiRNA into Breast Cancer Cells In Vitro and In Vivo
Source: J Funct Biomater. 2020 Sep 10;11(3):63. doi: 10.3390/jfb11030063 (PMC7565062; doi:10.3390/jfb11030063)
Supplement: Supplementary file 1 [file jfb-11-00063-s001.docx]

Supplementary Materials: Carbonate Apatite and Hydroxyapatite Formulated with Minimal Ingredients to Deliver SiRNA into Breast Cancer Cells In Vitro and In Vivo

Rowshan Ara Islam, Hamed Al-Busaidi, Rahela Zaman, Syafiq Asnawi Zainal Abidin,
Iekhsan Othman and Ezharul Hoque Chowdhury *

Jeffrey Cheah School of Medicine and Health Sciences, Monash University Malaysia, Jalan Lagoon Selatan, Bandar Sunway, Subang Jaya 47500, Selangor, Malaysia; Rowshan.Islam@monash.edu (R.A.I.); hamed.al-busaidi@monash.edu (H.A.-B.); rahela.zaman@monash.edu (R.Z.); syafiqnawi@gmail.com (S.A.Z.A.); iekhsan.othman@monash.edu (I.O.)

***** Correspondence: md.ezharul.hoque@monash.edu; Tel.: +603-5514-4978; Fax: +603-5514-6323

| 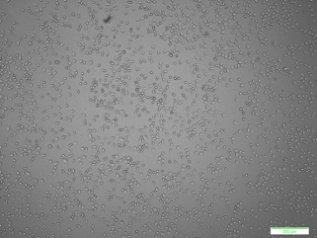 | 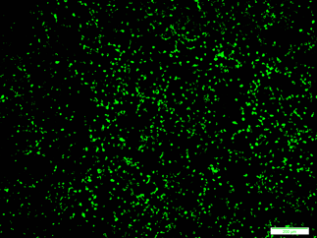 | 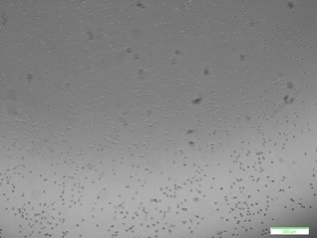 | 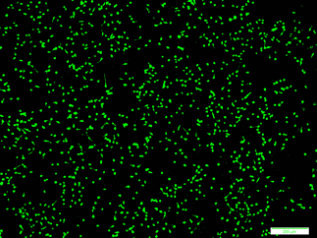 |
| --- | --- | --- | --- |
| Free siRNA in DMEM | Free siRNA in DMEM | CA-siRNA in DMEM | CA-siRNA in DMEM |
| 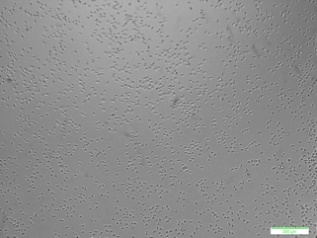 | 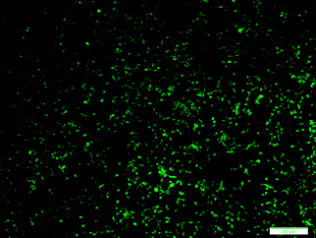 | 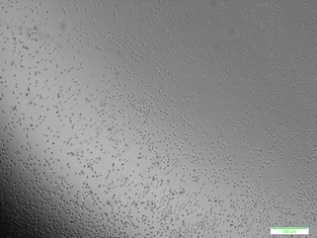 | 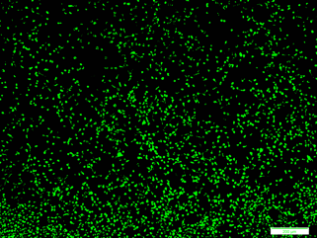 |
| Free siRNA in DMB | Free siRNA in DMB | CA-siRNA in DMB | CA-siRNA in DMB |
| 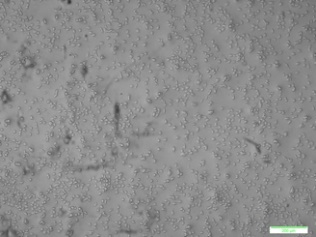 | 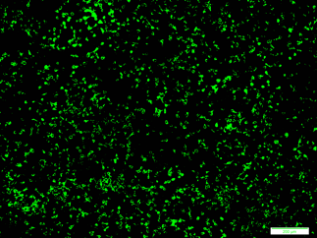 | 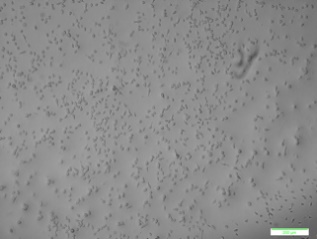 | 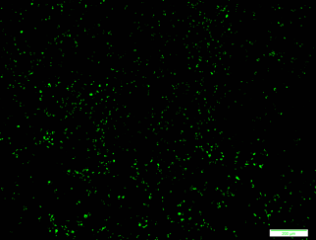 |
| Free siRNA in HBS | Free siRNA in HBS | HA-siRNA | HA-siRNA |

**Figure S1. Cellular uptake of NP bound Alexa Fluor 488 neg siRNA.** All NPs were prepared in 100 μL with 10 nM of siRNA and topped up to 1 mL with C-DMEM. After 4 h, cells were washed with 5-mM EDTA and PBS before viewing under microscope. Row 1: CA-in-DMEM; Row 2: CA-in-DMB; Row 3: HA.

| HA-siRNA with serum | 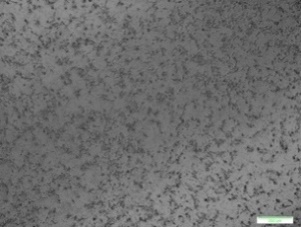 | 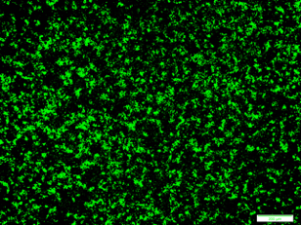 | 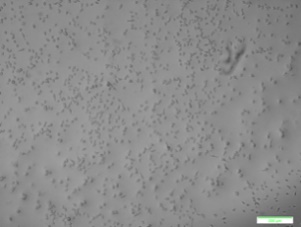 | 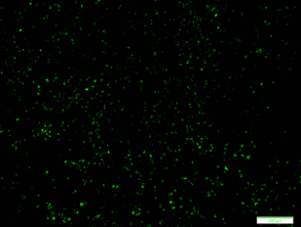 |
| --- | --- | --- | --- | --- |
|  | Before wash | Before wash | After wash | After wash |
| HA-siRNA without serum | 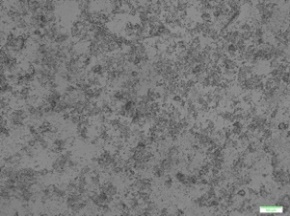 | 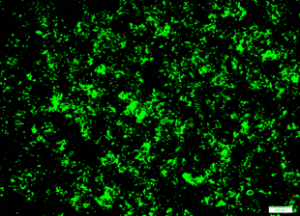 | 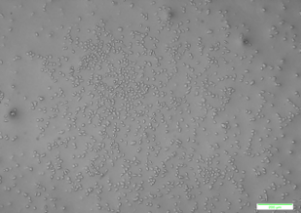 | 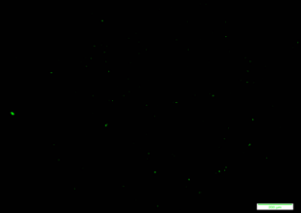 |
|  | Before wash | Before wash | After wash | After wash |

**Figure S2. Cellular uptake of HA-siRNA**. HA particles were prepared in 100 μL with 10 nM of siRNA and topped up to 1 mL with C-DMEM or serum free DMEM. After 4 h, cells were washed with 5-mM EDTA and PBS to remove extracellular HA. Images were taken before and after wash. Row 1: HA-siRNA with serum; Row 2: HA-siRNA without serum.
